# Insecticidal and Repellent Activity of *Siparuna guianensis* Aubl. (Negramina) against *Aedes aegypti* and *Culex quinquefasciatus*

**DOI:** 10.1371/journal.pone.0116765

**Published:** 2015-02-03

**Authors:** Raimundo Wagner Souza Aguiar, Suetonio Fernandes dos Santos, Fabricio da Silva Morgado, Sergio Donizeti Ascencio, Magnólia de Mendonça Lopes, Kelvinson Fernandes Viana, Julcemar Didonet, Bergmann Morais Ribeiro

**Affiliations:** 1 Universidade Federal do Tocantins, Departamento de Biotecnologia, Campus Universitário de Gurupi, Gurupi, Tocantins, Brazil; 2 Universidade Federal do Tocantins, Campus Universitário de Palmas, Tocantins, Brazil; 3 Universidade de Brasília, Departamento de Biologia Celular, Campus Universitário, Asa Norte, Brasília, Distrito Federal, Brazil; New Mexico State University, UNITED STATES

## Abstract

This study investigated the toxic effects of essential oils isolated from Siparuna guianensis against *Aedes aegypti, Culex quinquefasciatus* (eggs, larvae, pupae, and adult) and *Aedes albopictus* (C6/36) cells. The oviposition-deterring activity, egg viability, and repellence activity in the presence of different essential oils concentrations were determined. The essential oils showed high toxicity to all developmental stages of *A. aegypti* and *C. quinquefasciatus.* Furthermore, the oils also showed high repellent activity towards the adult stage of mosquitoes (0.025 to 0.550 μg/cm2 skin conferred 100% repellence up to 120 min) and in contact with cultured insect cells (C6/36) induced death possibly by necrosis. The results presented in this work show the potential of *S. guianensis* essential oils for the development of an alternative and effective method for the natural control of mosquitoes in homes and urban areas.

## Introduction

*Aedes aegypti* L. (Diptera: Culicidae) and *Culex quinquefasciatus* Say (Diptera: Culicidae) are insect vectors responsible for the transmission of several human diseases caused by protozoans (malaria), nematodes (lymphatic filariasis) and viruses (West Nile encephalitis, dengue fever, yellow fever and chikungunya fever). In 2010, there were more than 1.6 million cases of dengue virus infection in Latin America, and in 2013, 204,650 cases only in Brazil [1]. Dengue fever is a debilitating disease caused by a flavivirus and transmitted mainly by female *A*. *aegypti* mosquitoes. Because there are no vaccines available, the most effective way to prevent this disease is to control the insect vector. Filariasis is caused by the roundworm *Wuchereria bancrofti* (Cobb.), which is transmitted by *C*. *quinquefasciatus*. This parasite is endemic to many tropical regions, with an estimated 120 million individuals infected in 81 countries and more than 1 billion people are at risk of contracting the infection. In Brazil, the Metropolitan Region of Recife, located in the State of Pernambuco, has endemic areas of lymphatic filariasis, which constitutes a major challenge to public health [2].

In Brazil, chemical insecticides play an important role in the control of insect vectors [3]. However, the extended use of organochlorines, organophosphates, carbamates, and pyrethroids without any resistance management strategy has led to the emergence of several insecticide resistant populations. Therefore, there has been a resurgence of mosquito-borne diseases [4]. Chemicals derived from plants have been shown to function as general toxicants, growth and reproductive inhibitors, oviposition deterrents, fumigants for adult insects, and repellents. They can therefore provide an alternative for chemical repellent formulations used in mosquito control. [5, 6, 7].

*Siparuna guianensis* Aubl. (Family: Siparunaceae), also known as capitiú and negramina, is present in almost all of Brazil, but with greater frequency in Northern Brazil [8]. Chemical and ethnobiological studies on *S*. *guianensis* are limited, with only a few reports of traditional medicine in Central and South America, including the use of juice from leaves against fevers, and as a postpartum antibiotic [9]. In Brazil, *S*. *guianensis* is used in folk medicine and the leaves and flowers are considered carminative, aromatic, anti-dyspeptic, diuretic, and have stimulant and febrifuge properties [10]. However, no insecticidal activity has been reported for *S*. *guianensis* plants. Some plants (e.g., *Melia azedarach* L., *Sida acuta* Burm. f., *Hyptis suaveolens* (L.) Poit., *Acorus calamus* L., *Feronia limonia* L., *Andrographis paniculata* Burm.f.) have a natural toxicity against insect pests [11, 12, 13, 14, 15, 16]. In addition, essential oils from *Eucalyptus saligna* Sm. [17], *Pinus caribaea* Mor., *Pinus tropicalis* Mor. [18], and *Chenopodium ambrosioides* L. [19] have been shown to be toxic to mosquitoes. However, there are no thorough investigations into the insecticidal effects of the essential oils and their constituents from the leaf, stem, and fruit of *S*. *guianensis*. A recent study has shown that *S*. *guianensis* essential oil is also toxic to *Rhipicephalus microplus* also known as cattle-tick, an important tick that parasitize a variety of livestock species [20].

In this paper, we analyzed the chemical constituents of essential oils from the leaf, stem, and fruit of *S*. *guianensis* and tested their effects against *A*. *aegypti* and *C*. *quinquefasciatus*. We investigated the larvicidal, ovicidal, adulticidal, oviposition-deterring, and repellent effects of *S*. *guianensis* essential oil against *A*. *aegypti* and *C*. *quinquefasciatus in vivo* and its toxicity against C6/36 cells (*Aedes albopictus* Skuse).

## Materials and Methods

### Plant material and hydrodistillation

*Siparuna guianensis* was collected in Gurupi (11°43'45" latitude S, 49°04'07" longitude W) and Formoso do Araguaia (11°47'48" latitude S, 49°31'44" longitude W), Tocantins state, Brazil (The collections were approved by Conselho Nacional de Desenvolvimento Científico e Tecnológico, CNPq, nº 010580/2013–1). Taxonomic identification was confirmed by experts at the herbarium of the Federal University of Tocantins (Campus Porto Nacional), where samples were deposited with reference number 10.496. Essential oils of *S*. *guianensis* were extracted from the leaves, stem, and fruit by hydrodistillation in a Clevenger apparatus, as described by [21]. The present study did not involve endangered or protected species.

### Cells and insects

C6/36 *Aedes albopictus* cells were grown in Leibovitz L-15 medium (Gibco) supplemented with 5% fetal bovine serum (FBS) in standard adherent cell culture plates (Techno Plastic Products, Trasadingen, Switzerland), at 28°C. The colonies of *A*. *aegypti* and *C*. *quinquefasciatus* were originally established from insects collected from the field in the state of Tocantins, Brazil, (11°40'55.7" latitude S, 49°04'3.9" longitude W), where no insecticides have been used for the control of mosquitoes. Insects were maintained in the laboratory for at least five generations before being used in bioassays (The collections were approved by Conselho Nacional de Desenvolvimento Científico e Tecnológico, CNPq, nº 010580/2013–1).. All bioassays were conducted at 26 ± 1°C, 60.0 ± 5% RH, and a 12-h light and 12-h dark photoperiod. Fish food pellets (larvae), 10% honey solution (male adult) and rabbit blood (female adult) were used as food sources.

### C6/36 cell viability

In order to analyze the effect of essential oils of *S*. *guianensis* on the viability of mosquito cells in culture C6/36 cells from *A*. *albopictus* were incubated with serial dilutions of the essential oils of *S*. *guianensis* and cell viability was determined by the trypan blue exclusion method in the Countess Automated Cell Counter by Invitrogen (Carlsbad, CA, USA), using the manufacturer specified protocol. Adherent cells were plated at concentrations of 2 × 10^5^ cells/mL and incubated for 2 h at 28°C. The medium supernatant was replaced with new medium containing different concentrations of essential oil (0.86 to 0.086 µg/mL) mixed with dimethyl sulfoxide (DMSO). A negative control medium containing only DMSO was also prepared. The cells were then incubated at 28°C for 24 h and each treatment was performed in triplicate. After incubation, the cells were aspirated and an aliquot was mixed with equal amounts of trypan blue stain (0.4%). Cell viability was determined by the Countess Cell Counter using the following parameters: minimum size 7 µm, maximum size 30 µm, and circularity 80%. Statistical analysis to determine LC_50_ concentration was done with GraphPad Prism v6.04 (San Diego, CA, USA). Light microscopy evaluation (Axiovert 100; Zeiss, Oberkochen, Germany) was also carried out to observe the cytopathic effects induced by the essential oil.

### Chemical composition analysis of essential oils

The GC analysis of *S*. *guianensis* essential oils was performed using a Chemito 8510 GC (Chemito Technologies Pvt. Ltd, Mumbai, India) instrument equipped with a data processor. A BP-5 wide-bore capillary column (30 m × 0.53 mm i.d., 1.0-mm film thickness) was used for separation of the sample components (sample size 0.03 mL, measured using a Hamilton GC syringe with a 1.0-mL cap.). Hydrogen was used as the carrier gas at a flow rate of 5 mL/min and 20 psi inlet pressure. The GC column oven temperature went from 70°C to 210°C at a rate of 2.5°C/min, with a final hold time of 5 min. Both injector and detector (FID) temperatures were maintained at 230°C. GC-MS analysis was carried out on a Trace DSQ MS (Thermo Electron Corporation, Waltham, MA, USA), using a BP-5 capillary column (30 m × 0.25 mm × 0.25 μm), with helium as the carrier gas at a flow rate of 1 mL/min; split ratio 1:20. The column temperature went from 65°C to 210°C (10-min hold) at 3°C/min. Mass spectra were recorded in the range of 40–650 amu, operating at 70 eV, and the ion source temperature was maintained at 200°C. The constituents of the oil were identified using standard reference compounds and by matching the mass spectra fragmentation pattern with NIST Mass Spectra Library stored in the GC-MS database.

### Ovicidal test analysis

Fifty *A*. *aegypti* eggs were exposed (for 24h) to three concentrations (0.5, 1.0, and 2.0 µg/mL in 500 µL of DMSO and 29.5 mL of distilled water) of *S*. *guianensis* essential oils, that were chosen from serial dilutions that had minimum and maximum effects on egg hatching. Each test was replicated ten times. Distilled water mixed with 500 µL DMSO served as the control. In the case of *C*. *quinquefasciatus*, one egg raft was used for each replication. The percentage of egg viability was calculated by the following formula: %V = (T—I)/T × 100, where %V is the percentage egg viability, T is the number of viable eggs in control treatment without application of essential oil, and I is the number of viable eggs after treatment with essential oil.

### Mosquito ovipositor-deterrence test analysis

The effect of *S*. *guianensis* essential oils on egg-laying by *A*. *aegypti* and *C*. *quinquefasciatus* was carried out according to the methodology previously described [12]. Twenty-five gravid females fed on rabbit blood (1–7 d old) and fifty males were simultaneously exposed, in an entomological cage (35 cm width x 23 cm depth x 47 cm length), to ovitraps containing different extract concentrations or controls and incubated at 28 ± 2°C. The cages contained six replicates in plastic cups (100 mL) containing 0.5, 1.0, 1.5, and 2.0 µg of essential oil in 500 μL of DMSO and a control with tap water and 500 µL of DMSO. The test was repeated four times. The numbers of eggs and egg rafts were counted on the seventh day after treatment. The percentage of oviposition inhibition was calculated as previously described [22].

### Larvicidal and pupicidal activity assay

Initially, we tested the toxicity of the essential oils obtained from the stem, fruit, and leaf of *S*. *guianensis* against the fourth instar larvae of *A*. *aegypti* and *C*. *quinquefasciatus*, according to the methodology previously described [23]. Because the highest yield and toxicity was obtained from the leaves, we chose *S*. *guianensis* leaf essential oil for further assays. Toxicity tests for essential oil were conducted against first, second, third, and fourth instar larvae and pupae. Twenty-five insects were placed in 100-mL cups with 29.5 mL of degassed distilled water, and 500 µL of DMSO solution containing the essential oil of *S*. *guianensis*. For first and second larvae, we used 0.10–0.95 µg/mL of essential oil and 0.50–2.50 µg/mL for the third and fourth instar larvae and pupae. Each cup was left at room temperature. The control was treated as above but without essential oil. Each treatment was done with three replicates. Toxicity was reported as LC_50_ and LC_95_, representing the concentrations in µg/mL that killed 50 and 95% of mosquitoes.

### Adult toxicity assay

The essential oil of *S*. *guianensis* was dissolved in acetone to reach the desired concentrations (0.20–0.80 µg/mL). An aliquot (0.50 mL) of the test solution was dispensed in glass bottles of 50 mL, with an internal area of 60 cm^2^. The solvent was allowed to evaporate at room temperature and the essential oil of *S*. *guianensis* stayed in the inner wall of the glass bottle [11]. Fifty female *A*. *aegypti* and *C*. *quinquefasciatus* adults were then introduced into the glass bottle and insect mortality (LC_50_ and LC_95_) was recorded 1 h after the treatment. The experiment was repeated three times.

### Time response assay

For each developmental phase of *A*. *aegypti* and *C*. *quinquefasciatus*, insect mortality was initially estimated by performing concentration response curves. Mortality was recorded after 0.5–55 min of exposure to essential oil of *S*. *guianensis* and the response time (LT) activity was reported as LT_50_ and LT_95_, in min.

### Repellent activity assay

The repellent activity and RD_50_ of the essential oil of *S*. *guianensis* was performed according to the methodology described by [24, 25]. One hundred blood-starved *A*. *aegypti* and *C*. *quinquefasciatus* female insects, 4–5 days old, were kept in a net cage (24 × 24 × 24 cm^3^). The arms of the person conducting the test were cleaned with ethanol. After air-drying, 25 cm^2^ of the dorsal side of the skin on each arm was exposed, and the remaining area was covered by rubber gloves. *S*. *guianensis* essential oil was dissolved in ethanol, while ethanol alone served as a negative control and DEET (14.55%), present in a commercial product, as a positive control. The essential oil of *S*. *guianensis* at a concentration of 0.025–0.550 µg/cm^2^ was applied to one arm and the control and treated arms were introduced simultaneously into the cage. The numbers of bites were counted over 10 min every 120 min, from 4 p.m. to 6 p.m. The experiment was repeated five times. The percentage of protection was calculated by using the following formula, as previously described [26]:

P=(Nc−Nt)/Ncx100 (1)

Where P (%) is the percentage of protection, Nc is the number of bites received by the control arm, and Nt is the number of bites received by the treated arm.

### Statistical analysis

LC and LT were determined by probit analysis [27]. Mortality data were corrected using the Abbott’s formula [28]. Significant differences between values were determined by using analysis of variance followed by Tukey’s test (P<0.01 and P<0.05). Statistical analysis was performed using SISVAR 4.6 [29], and graphs were produced using SIGMA PLOT 11.0 (Systat Software, Inc. San Jose, USA).

## Results and Discussion

Essential oils purified from the leaves, stem, and fruit of *S*. *guianensis* were analyzed by GC and GC-MS and their qualitative and quantitative compositions were determined (Table 1). The main components of oils from the leaves were *β*-myrcene (79.71%) and 2-undecanone (14.58%). From the stem, the major components were *β*-myrcene (26.91%), δ-elemene (20.92%), germacrene D (9.42%), α-limonene (7.91%), and bicyclo-germacrene (7.79%). The main components in the fruit were 2-tridecanone (38.75%), 2-undecanone (26.5%) and *β*-myrcene (16.42%).

**Table 1:** Chemical composition, concentrations (%), and Kovats indices for *Siparuna guianensis* essential oil.

Compound	Concentrations (%)			Ric*
	Leaf	Stem	Fruit	
Santolina triene	t^2^	-	-	927
2-Tridecanone	­	-	38.75	1398
2-Undecanone	14.58	12.8	26.5	1276
3-carene	-	1.36	-	1007
*α*-cadinol	-	-	1.05	1653
Agarospirol	-	-	3.85	1631
*α*-limonene	-	7.91	-	1828
Bicyclo-germacrene	1.21	7.79	-	1493
Cadinol	-	1.42	-	1653
Camphene	0.11	-	-	956
D-limonene	0.67	-	2.87	1017
Epi-*α*-cadinol	0.18	-	-	1644
Spathulenol	0.25	6.09	-	1574
Germacrene D	0.80	9.42	-	1480
Germacrene A	0.42	-	-	1504
Germacrene B	0.11	-	-	1556
Terpinolene	0.16	-	-	1089
*α*-cadinol	0.12	-	-	1653
*α*-caryophyllene	-	1.31	-	1419
*α*-copaene	0.13	-	-	1375
*α*-pinene	0.38	-	3.06	923
*β*-caryophyllene	0.35	-	-	1418
*β*-elemene	0.36	1.36	-	1389
*β*-myrcene	79.71	26.91	16.42	990
Nonanol	-	-	5.45	1154
*β*-ocimene	0.64	-	-	1049
*β*-pinene	0.17	-	-	984
*γ*-cadinene	0.09	-	-	1516
δ-elemene	-	20.92	-	1435
Non identified (%)	-	2.71	2.05	
Total identified (%)	100	97.29	97.95	

*Ric = retention index calculated;

^2^t = traces (<0.1%).

The observed concentration of the main constituents in the essential oils of *S*. *guianensis* was different from that reported by [20, 30, 31], suggesting a considerable variability in the oil samples studied. Essential oils from *S*. *guianensis* samples collected in different Amazon regions and Minas Gerais Estate were shown to have variable amounts of major compounds [32, 20]. These changes in the composition of essential oils might arise from several environmental (climatic, seasonal, or geographic) and genetic differences, since the specimen analyzed was collected from a region of the Brazilian cerrado (a savannah-like area), which is a completely different biome when compared with the Amazon.

Our analysis identified mainly sesquiterpene hydrocarbons, oxygenated sesquiterpenes and monoterpene hydrocarbons in the essential oils of the fresh leaves, stems and fruits of *S*. *guianensis*.

**Figure 1:**
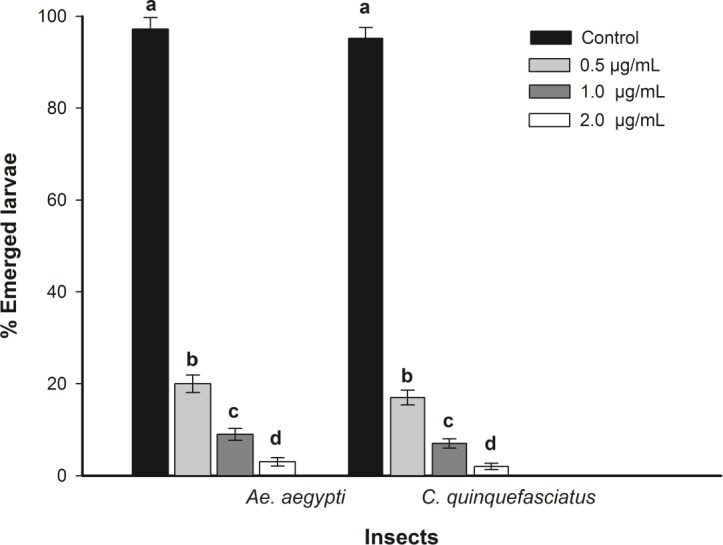
Ovicidal activity of *S*. *guianensis* (negramina) essential oil on the emergence of *A*. *aegypti* and *C*. *quinquefasciatus* larvae. Each datum represents the mean of four replicates. Means followed by different letters are significantly different (P<0.01) by Tukey’s test.

Essential oils purified from *S*. *guianensis* dramatically influenced the viability of *A*. *aegypti* and *C*. *quinquefasciatus* eggs (Fig. 1). At a concentration of 0.5 µg/mL, less than 20% of egg hatching was observed for both insects (Fig. 1). For concentrations of 1.0 and 2.0 µg/mL, only 5% of egg hatching was observed for both insects (Fig. 1). Other plant essential oils and/or leaf extracts have also shown toxicity to mosquito eggs. *Pinus caribaea* and *Pinus tropicalis* essential oils and the leaf extract from *Chenopodium ambrosioides* have been shown to be toxic to to *A*. *aegypti* eggs [18] and *C*. *quinquefasciatus* egg rafts [19], respectively.

**Figure 2:**
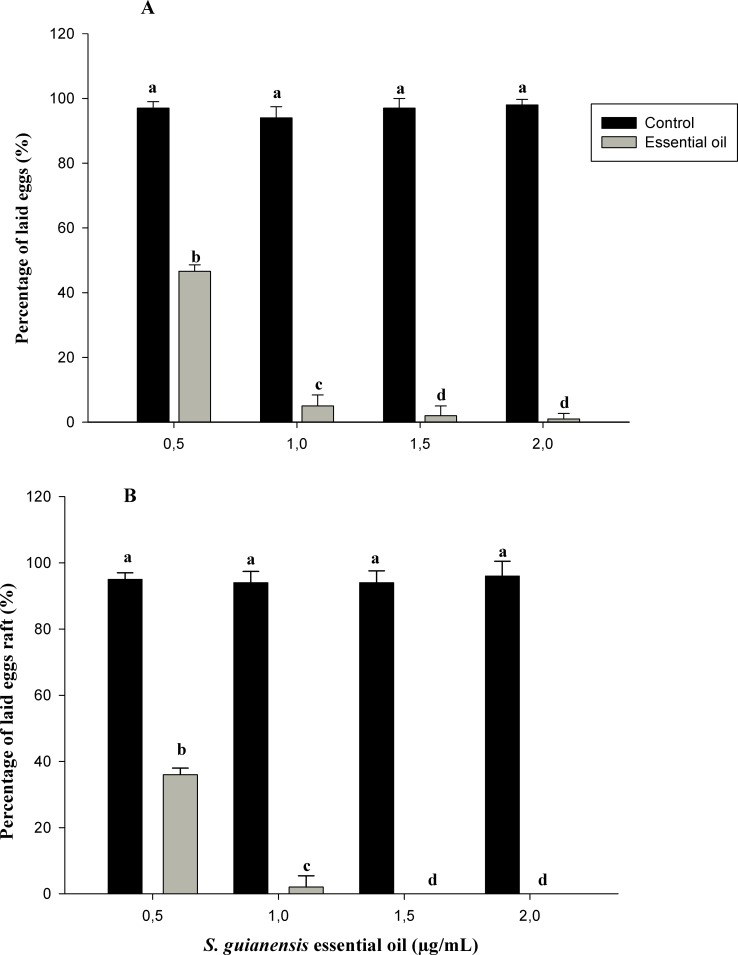
Average proportion of eggs and egg rafts laid in ovitraps of *A*. *aegypti* (A) and *C*. *quinquefasciatus* (B) treated with different concentrations of essential oils of *S*. *guianensis*. Control: Number of laid eggs ovitraps treated with water + DMSO. Different letters between columns indicate significant differences. Means followed by different letters are significantly different (P<0.01). Analysis of variance followed by Tukey’s test.

We found that the essential oils also proved to be deterrents to oviposition in both species of mosquitoes. The amount of *A*. *aegypti* and *C*. *quinquefasciatus* eggs lain in ovitraps treated with different concentrations of essential oil of *S*. *guianensis* was lower than that in controls (Fig. 2A and 2B). Oviposition-inhibiting effects against *A*. *aegypti* and *C*. *quinquefasciatus* have also been observed for other plant essential oils [11, 12, 33].

Stem, leaf, and fruit essential oils of *S*. *guianensis* showed high toxicity against the fourth instar *A*. *aegypti* and *C*. *quinquefasciatus* larvae (Table 2). The LC_50_ values obtained for stem, leaf, and fruit essential oils were 1.76, 0.98, and 2.46 µg/mL against *A*. *aegypti* and 1.36, 0.89, and 2.45 µg/mL against *C*. *quinquefasciatus*, respectively. The leaf essential oil showed the lowest LC_50_ towards fourth instar larvae of *A*. *aegypti* and *C*. *quinquefasciatus*, so we chose this extract to perform the toxicity tests for all insect stages (egg, larval, pupal, and adult).

**Table 2:** LC_50_ and LC_95_ values of the essential oil from different plant parts of *S*. *guianensis* against fourth instar larvae of *A*. *aegypti* and *C*. *quinquefasciatus*.

Part	A. aegypti		C. quinquefasciatus	
	LC_50_ (µg/mL)	LC_95_ (µg/mL)	LC_50_ (µg/mL)	LC_95_ (µg/mL)
Stem	1.76b	2.44b	1.36b	2.36 b
Leaf	0.98a	1.46a	0.89a	1.21 a
Fruit	2.46c	3.76c	2.45c	3.45 c

Each datum represents the mean of six replicates. Means followed by different letters are significantly different (P<0.05). Analysis of variance followed by Tukey’s test.

Toxicity of the *S*. *guianensis* essential oil against the fourth instar *A*. *aegypti* and *C*. *quinquefasciatus* larvae were higher than those from essential oils derived from the fruit of *Juniperus macropoda* and seeds of *Pimpinella anisum* [11]. The LD_95_ values of both species were 171.3 and 115.7 µg/mL against *A*. *aegypti* and 204.8 and 149.7 µg/mL against *C*. *quinquefasciatus*, respectively. Leaf and stem essential oils of *Chloroxylon swietenia* against *A*. *aegypti* [34] showed LD_50_ values of 16.5 and 20.4 µg/mL, respectively. The larvicidal effect of *S*. *guianensis* essential oil was also superior to that found by [23], who used essential oils of *Eucalyptus camaldulensis* and *Eucalyptus urophylla* against *A*. *aegypti* and *A*. *albopictus*. Their LC_50_ values were 95.5 and 31.0 µg/mL against *A*. *aegypti* and 295.8 and 55.3 µg/mL against *A*. *albopictus* for *E*. *camaldulensis* and *E*. *urophylla*, respectively.

The LC_50_ and LC_95_ values of *S*. *guianensis* essential oil differ depending on the stage of development of mosquitoes (Table 3). We found that the LC_50_ of *S*. *guianensis* essential oil against the pupae of *A*. *aegypti* (LC_50_: 1.69 µg/mL) and *C quinquefasciatus* (LC_50_: 1.31 µg/mL) was higher than that of all larval stages (Table 3). The essential oil was more toxic to younger larvae of *A*. *aegypti* and *C*. *quinquefasciatus*. The LC_50_ values for the first instar *A*. *aegypti* and *C*. *quinquefasciatus* larvae were 0.21 µg/mL and 0.31 µg/mL, respectively (Table 3). This is higher than the LC_95_ value of a synthetic insecticide (temephos) (0.072 mg/L^-1^) against *C*. *quinquefasciatus* third instar larvae [3]. A previous study showed that temephos has an LC_50_ value of 0.0023 µg/mL towards third instar susceptible *A*. *aegypti* larvae, which is lower than the LC_50_ of the *S*. *guianensis* essential oil (0.75 µg/mL) [35]. Nevertheless, isolation and identification of larvicidal components in *S*. *guianensis* essential oil could lead to the development of alternative compounds to replace synthetic insecticide [36]. Besides insects, *S*. *guianensis* essential oils were recently shown to be toxic to the tick *R*. *Microplus* [20].

**Table 3:** LC_50_ and LC_95_ values of *S*. *guianensis* essential oils against different developmental stages of *A*. *aegypti* and *C*. *quinquefasciatus*.

Insect	Stage	Slope ±SEM	LC_50_ (µg/mL)	FI (LC_50_)	LC_95_ (µg/mL)	FI (LC_95_)	χ^2^	P
A. aegypti	1 instar	0.81±0.04	0.21d	0.12–0.32	0.45d	0.36–0.51	4.78	0.49
	2 instar	0.84±0.03	0.55b	0.41–0.75	0.80c	0.67–0.88	3.24	0.05
	3 instar	0.94±0.06	0.75b	0.51–0.92	0.85c	0.71–0.94	4.24	0.02
	4 instar	0.94±0.02	0.91b	0.73–1.02	1.48b	0.96–1.82	2.21	0.11
	Pupae	0.60±0.07	1.69a	1.30–1.90	2.15a	1.88–2.51	4.15	0.11
	Adult	0.70±0.11	0.38c	0.33–0.48	0.65c	0.55–0.91	4.02	0.21
C. quinquefasciatus	1 instar	0,49 ± 0.05	0.31e	0.22–0.36	0.56d	0.47–0.59	2.78	0.29
	2 instar	0.62 ± 0.07	0.42d	0.39–0.46	0.75c	0.62–0.82	2.24	0.15
	3 instar	0.75 ± 0.05	0.59c	0.55–0.62	0.85c	0.71–0.94	4.65	0.12
	4 instar	0.60 ± 0.10	0.78b	0.67–0.82	0.99b	0.96–1.12	2.78	0.11
	Pupae	1.14 ± 0.18	1.31a	1.10–1.50	2.22a	1.98–2.51	3.17	0.08
	Adult	1.04 ± 0.12	0.39d	0.37–0.46	0.60d	0.50–0.81	4.21	0.19

SEM = standard error of the mean; LC = lethal concentration (µL/mL); FI = fiducial interval at 95% of probability; χ^2^ = Chi squared. Means followed by different letters are significantly different (P<0.05). Analysis of variance followed by Tukey’s test.

The essential oil of *S*. *guianensis* LT_50_ and LT_95_ were obtained using the LC_95_ concentrations determined for each stage of *A*. *aegypti* and *C*. *quinquefasciatus* (Table 4). The time required to achieve 95% mortality (LT_95_) was 40.97 min for the pupae of *C*. *quinquefasciatus* and 45.79 min for pupae of *A*. *aegypti* (Table 4). However, for the larval and adult stages, it took less than 33 min to kill 95% of both stages. These results could be associated with the amount of essential oil that can penetrate the pupae when compared with the larval and adult stages or to differences in metabolic detoxification [36].

**Table 4:** LT_50_ and LT_95_ values of *S*. *guianensis* (negramina) essential oils against different developmental stages of *A*. *aegypti* and *C*. *quinquefasciatus* using the LC_95_.

Insects	Stages	Slope ±SEM	LT_50_ (Min)	FI (LT_50_)	LT_95_ (Min)	FI (LT_95_)	χ^2^	P
A. aegypti	1 instar	0.84 ± 0.09	4.38e	2.92–5.12	6.42e	5.81–7.06	4.47	0.37
	2 instar	0.70 ± 0.02	6.62d	5.30–7.14	8.05d	7.27–10.67	3.32	0.10
	3 instar	0.71 ± 0.12	12.11c	8.20–14.06	18.85c	16.03–22.70	4.32	0.09
	4 instar	0.64 ± 0.14	20.32b	16.40 –23.02	28.20b	23.09–34.04	6.07	0.17
	Pupae	0.58 ± 0.04	30.78a	25.99 –34.41	45.79a	42.09–56.45	1.99	0.40
	Adult	0.49 ± 0.10	6.78d	5.49–7.43	9.68d	7.59–12.29	3.93	0.22
C. quinquefasciatus	1 instar	0.84 ± 0.09	4.78e	3.78–5.51	6.78d	6.22–7.39	3.27	0.27
	2 instar	1.11 ± 0.12	7.92d	6.90–8.34	9.52c	8.21–11.56	4.12	0.13
	3 instar	1.02 ± 0.12	13.51c	11.20 –15.06	20.23b	18.21–23.70	4.52	0.19
	4 instar	0.85 ± 0,14	21.47b	19.40 –24.02	43.12a	38.09–47.02	5.17	0.18
	Pupae	0.91 ± 0.04	32.78a	31.22 –36.41	40.97a	39.22–54.21	2.91	0.25
	Adult	0.76 ± 0.09	5.45e	4.92 –.6.13	8.06c	7.59–9.29	2.98	0.29

LT = response time (µL/mL); FI = fiducial interval at 95% of probability; χ^2^ = Chi squared. Means followed by different letters are significantly different (P<0.05). Analysis of variance by the Tukey test.

Besides its toxicity, the essential oil of *S*. *guianensis* shows promise as a repellent (Table 5). The doses to repel 50% of the adult mosquito population (RD_50_) were 0.438 and 0.662 µg/cm^2^ for *A*. *aegypti* and *C*. *quinquefasciatus*, respectively. Repellent values of 0.450 to 0.550 µg/cm^2^ obtained 100% repellency for *A*. *aegypti* and *C*. *quinquefasciatus* for up to 120 min (Table 6). From these results, the essential oil of *S*. *guianensis* has the potential to be an excellent candidate for a mosquito-repellent formulation. Furthermore, the repellent activity obtained by the essential oil concentration above 0.450 µg/cm^2^ was higher than that of a commercial product usually sold in Brazil as an insect repellent (with *N*,*N*-diethyl-*m-*toluamide [DEET] 14.55% as the active ingredient), which was used as a positive control, and had a maximum protection of 30 min (Table 6).

**Table 5:** Repellent activity of *S*. *guianensis* essential oil against *A*. *aegypti* and *C*. *quinquefasciatus*.

Insect	Slope ± SEM	RD_50_	FI(RD_50_)	RD_95_	FI (RD_95_)
A. aegypti	1.12 ± 0.09	0.438a	2.92–6.70	0.642a	5.81–7.72
C. quinquefasciatus	0.92 ± 0.06	0.662a	4.90–8.14	0.805a	5.37–10.67

RD_50_ 0 repellency dose (in µg/cm^2^ of skin) repels 50% of *A*. *aegypti* and *C*. *quinquefasciatus*; RD_95_ repellency dose (in µg/cm^2^ of skin) repels 95% of *A*. *aegypti* and *C*. *quinquefasciatus*.

**Table 6:** Protective activity of *S*. *guianensis* essential oil against *C*. *quinquefasciatus* and *A*. *aegypti*.

Insect	Concentration of essential oil (µg/cm^2^/skin)	% of repellency (±SE)				
		Time post application of repellent (min)				
		5	15	30	60	120
A. aegypti	0.025	100 ± 0	100 ± 0	72 ± 2.2	46 ± 4.5	30 ± 5.0
	0.250	100 ± 0	100 ± 0	100 ± 0	57 ± 5.0	52 ± 7.5
	0.450	100 ± 0	100 ± 0	100 ± 0	81 ± 5.0	72 ± 4.0
	0.550	100 ± 0	100 ± 0	100 ± 0	100 ± 0	100 ± 0
	DEET	100 ± 0	100 ± 0	100 ± 0	82 ±1.80	60 ± 2.10
C. quinquefasciatus	0.025	100 ± 0	100 ± 0	100 ± 0	55 ± 2.5	30 ± 3.6
	0.250	100 ± 0	100 ± 0	100 ± 0	48 ± 4.3	42 ± 3.5
	0.450	100 ± 0	100 ± 0	100 ± 0	100 ± 0	100 ± 0
	0.550	100 ± 0	100 ± 0	100 ± 0	100 ± 0	100 ± 0
	DEET*	100 ± 0	100 ± 0	100 ± 0	90 ± 3.1	80 ± 2.0

Values are mean of five replications ± SE

*DEET = *N*,*N*-diethyl-*m-*toluamide at 14.55%

The compounds 2-undecanone and 2-tridecanone, which are major components of the essential oil of *S*. *guianensis*, have been shown to be involved in the resistance of tomatoes to herbivorous pests [37] and in repellence to blood-feeding insects [38]. In this context, repellent properties of the essential oil of *S*. *guianensis* can be associated with oxygenated compounds, as shown in Table 1.

**Figure 3:**
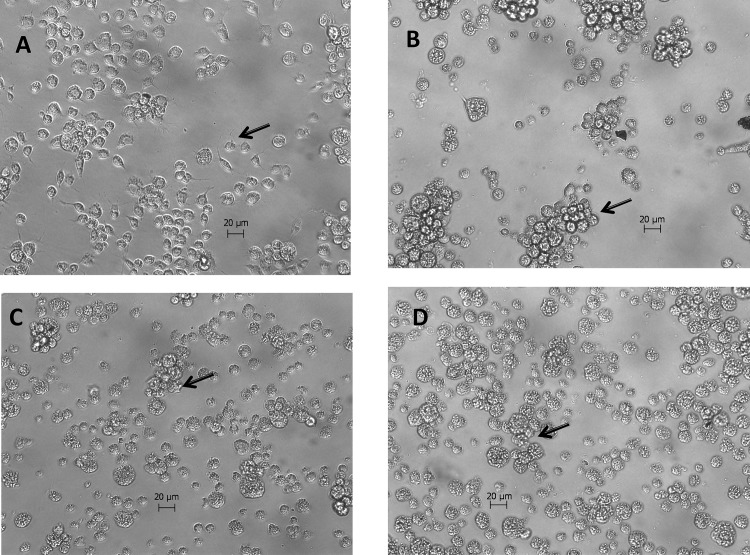
Light micrographs of C6/36 cells of *A*. *albopictus* treated with essential oil of *S*. *guianensis* after 2 h. A: C6/36 cells treated with DMSO + medium. B, C, and D: C6/36 cells treated with essential oil of *S*. *guianensis* at concentrations 0.2, 0.4, and 0.8 µg/mL, respectively.

We also analyzed the *in vitro* toxicity of essential oil of *S*. *guianensis* on C6/36 cells derived from *A*. *albopictus*. As observed by light microscopy (Fig. 3), the cytopathic effect of the essential oil of *S*. *guianensis* can be associated with the loss of plasma membrane integrity, leading to cell death by necrosis in a dose-dependent manner. As observed in Fig. 3, a concentration 0.86 µg/mL *S*. *guianensis* essential oil lowered cell viability of the C6/36 cells to less than 20%, compared with 90% in the control (S1 Fig.). The LC_50_ determined to be 0.15 µg/mL.

This study showed that *S*. *guianensis* essential oil has significant ovicidal, larvicidal, pupicidal, and adulticidal effects as well as repellent activity against *A*. *aegypti* and *C*. *quinquefasciatus*. Furthermore, we showed a high cytotoxic effect on *A*. *albopictus* C6/36 cells. Considering the wide distribution of *S*. *guianensis* throughout neotropical areas of the world and the growing resistance of *A*. *aegypti* and *C*. *quinquefasciatus* populations to synthetic organic pesticides [3, 36], *S*. *guianensis* could be further studied as a source of potential alternative strategies for controlling and repelling insect vectors.

## Conclusion

The essential oil of *S*. *guianensis* is highly toxic to all developmental stages of *A*. *aegypti* and *C*. *quinquefasciatus*. Furthermore, it has a high mosquito repellent activity. These properties of the *S*. *guianensis* essential oil demonstrate its potential for use as a natural insecticide against mosquitoes.

## Supporting Information

S1 Fig Viability of C6/36 cells from *A*. *albopictus* treated with different concentrations of essential oils of *S*. *guianensis*. (TIF)
