# Supplementary figures and images for: Insecticidal and Repellent Activity of Siparuna guianensis Aubl. (Negramina) against Aedes aegypti and Culex quinquefasciatus
Source: PLoS One. 2015 Feb 3;10(2):e0116765. doi: 10.1371/journal.pone.0116765 (PMC4315403; doi:10.1371/journal.pone.0116765)

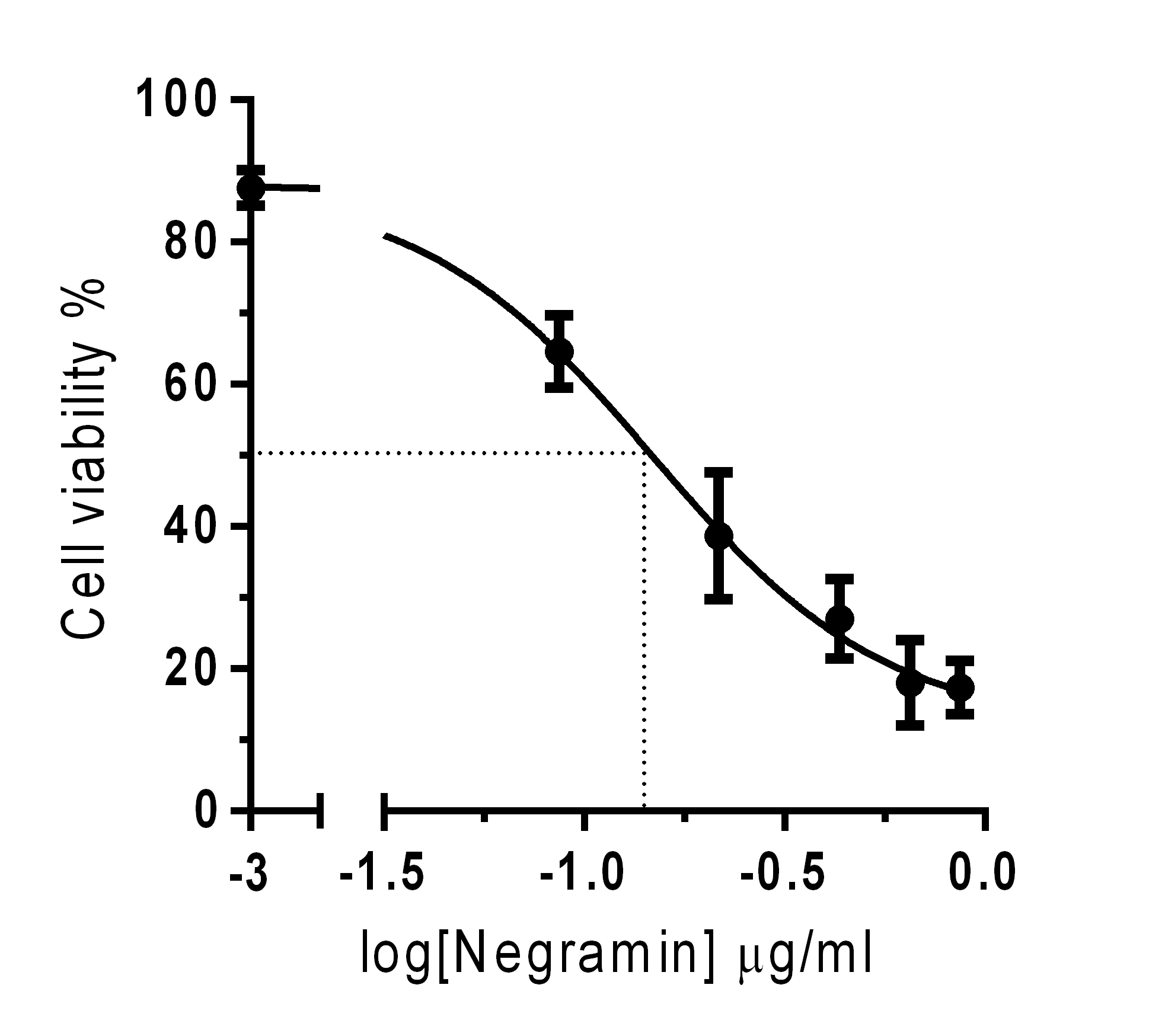

Supplement: S1 Fig — (TIF) [file pone.0116765.s001.tif]
